# Trait‐independent habitat associations explain low co‐occurrence in native and exotic birds on a tropical volcanic island

**DOI:** 10.1002/ece3.10322

**Published:** 2023-07-20

**Authors:** Jean‐Yves Barnagaud, Olivier Flores, Gérard Balent, Jacques Tassin, Luc Barbaro

**Affiliations:** ^1^ CEFE, Univ Montpellier, CNRS, EPHE‐PSL University Montpellier France; ^2^ Univ de La Réunion, UMR PVBMT Saint‐Pierre France; ^3^ DYNAFOR, University of Toulouse, INRAE Castanet‐Tolosan France; ^4^ CIRAD, UPR Forêts & Sociétés Montpellier France; ^5^ CESCO, Museum National d'Histoire Naturelle, CNRS, Sorbonne University Paris France

**Keywords:** ecological traits, habitat changes, insular birds, Mascarenes, oceanic islands, species introductions

## Abstract

On oceanic islands, strong human impacts on habitats, combined with introductions of exotic species, modify the composition of terrestrial bird assemblages and threaten their ecological functions. In La Réunion, an oceanic island located in the Madagascan region, a national park was established in 2007 to counter the ecosystem‐level effects of three centuries of habitat conversion, native species destruction and exotic species introductions. Here, we investigated how bird assemblages were structured in these human‐modified landscapes, 10 years before the national park set out its first conservation measures. We used a combination of multivariate statistics and generalized additive models to describe variations in the taxonomic and functional composition and diversity of 372 local bird assemblages, encompassing 20 species, along gradients of habitat composition and configuration. We found that native species were tied to native habitats while exotic species were associated with urban areas and man‐modified landscape mosaics, with some overlap at mid‐elevations. Species' trophic preferences were segregated along habitat gradients, but ecological traits had an overall weak role in explaining the composition of species assemblages. Hence, at the time of the survey, native and exotic species in La Réunion formed two spatially distinct species assemblages with contrasting ecological trait suites that benefited from antagonistic habitat compositions and dynamics. We conclude that our results support the analysis of historical data sets to establish reference points to monitor human impacts on insular ecosystems.

## Graphical abstract

We investigated bird assemblages in La Réunion island, Mascarenes. We showed how habitat separates native and introduced exotic bird species independently from their ecological traits. This study stands as a landmark for species‐habitat relationships prior to the establishment of a major protected area intended to the conservation of endemic ecosystems.

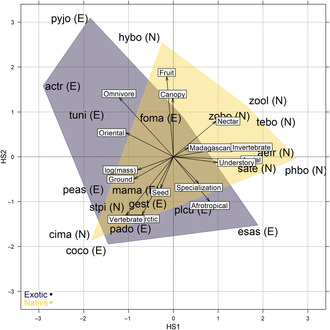

## 1 INTRODUCTION

The Madagascan region is the smallest and the second most distinctive of the world's 11 zoogeographic regions (Holt et al., 2013). Formed exclusively of oceanic islands spread throughout the western Indian Ocean, it is relatively species‐poor but displays disproportionately high endemism rates. For instance, 41% of the region's 382 native terrestrial bird species are regional endemics. This percentage even rises to 75% in La Réunion, the second largest island of the region after Madagascar and the largest of the Mascarenes archipelago (Safford et al., 2015). However, assessments of human impacts on insular ecosystems are deficient in the Madagascan region, even on the best‐studied islands such as La Réunion, hampering the establishment and evaluation of initiatives for the conservation of insular biodiversity.

On oceanic islands, long‐term isolation favoured the evolution of ecosystems involving a low number of endemic species which support nonsubstitutable ecological functions, notably in birds (Boyer & Jetz, 2014; Kaiser‐Bunbury et al., 2010; Şekercioğlu et al., 2016). Birds' contributions to insular ecosystems have been particularly well investigated in islands of the Indo‐Pacific, such as Hawaii (Barton et al., 2021) or New Zealand (Anderson et al., 2021). Plant–bird interactions likely to support key ecological functions are also documented in the Madagascan region, although case studies remain local and scarce. For instance, in La Réunion, the pollination of the endemic orchid *Angraecum bracteosum* by the endemic Reunion olive White‐eye *Zosterops olivaceus* recalls the pollination of *Clermontia* (Lobelioideae: Campanulaceae) by endemic Hawaiian birds (Aslan et al., 2014; Micheneau et al., 2008). However, the extent to which these specialized plant‐bird interactions still sustain their ecosystem functions in spite of the human‐driven depletion of Madagascan avifaunas remains unknown (Albert et al., 2020; Anderson et al., 2021; Schleuning et al., 2014; Şekercioğlu et al., 2016).

The Madagascan region is sadly famous for its high extinction rates, especially in large frugivorous birds (Heinen et al., 2018). In La Réunion, the first human colonization occurred in the late 17th century, far later than in many other tropical archipelagos. It was accompanied by novel and intense pressures, which led many terrestrial bird species to severe range reductions or extinction. Since then, approximately two‐thirds of the island's native vegetation has been converted for agriculture or replaced by exotic vegetation (Lagabrielle et al., 2009; Mourer‐Chauvire et al., 1999). Rapid habitat destructions, on top of overhunting and other pressures, were likely the most critical cause for the extinction of all large vertebrates (Albert, Flores, Baider, et al., 2021), inducing ecosystem‐level cascading effects in a few decades or centuries (Mourer‐Chauvire et al., 1999; Strasberg et al., 2005; Whittaker & Fernández‐Palacios, 2007). In particular, many of the common fleshy‐fruited tree species on which frugivorous birds fed declined as lowland native forests were nearly eradicated (Albert et al., 2018). In turn, the extinction of frugivores, which sustained seed dispersal, amplified the decline of most fleshy‐fruit plant species in the 300 years after the first European settlement (Albert et al., 2020; Albert, Flores, Baider, et al., 2021). Conversely, the abundance and diversity of exotic plant species now peaks close to the middle of the elevation gradient (Tassin & Riviere, 2003), and wide extents of high‐altitude landscapes have been converted into pastures (Strasberg et al., 2005).

Island endemic birds are characterized by low dispersal abilities, failure to cope with the lack of native food resources in human modified habitats, and high mortality rates due to introduced mammalian predators such as cats or rats. Conversely, exotic species that establish feral populations on islands unsurprisingly exhibit ecological traits associated with ecological opportunism, use of human resources and ability to colonize novel environments (Blackburn et al., 2009; Cassey, 2002; Duncan et al., 2003). Accordingly, the propensity of exotic bird species to invade islands from their initial introduction locations depends on an interaction between the invasibility of nonconverted native habitats and species' propensity for colonization, determined by their ecological traits and the conditions of their introduction (Alpert et al., 2000; Blackburn et al., 2009). The introduction of exotic species has in some instances compensated, or even exceeded in number, the man‐mediated extinction of native and endemic species (Sax et al., 2002; Whittaker et al., 2014). However, native and exotic bird species exhibit low functional redundancy, and are segregated among habitats along elevational and anthropogenic disturbance gradients (Barnagaud et al., 2014, 2022; Fischer & Lindenmayer, 2007; Whittaker & Fernández‐Palacios, 2007).

Because of their trait and habitat differences, exotic species do not usually replace native species' lost ecological functions (Anderson et al., 2021; Duncan et al., 2003; Vila et al., 2011), except in idiosyncratic situations such as the replacement of *Clermontia*'s native pollinators by introduced *Zosterops* in Hawaii (Aslan et al., 2014, but see Pejchar, 2015 for a contrasting example on seed dispersal also in Hawaii). Hence, although bird introductions increase taxonomic diversity (the diversity of species) and functional diversity (the diversity of ecological traits) at the island scale (Whittaker et al., 2014), their effect on the composition of local species assemblages and ecosystem functions remains unclear. For instance, among the 20 exotic bird species established in La Réunion since the 19th century, the Red‐whiskered Bulbul (*Pycnonotus jocosus*) is the only one known to enter native forests, thus liable to modify the composition of native plant assemblages through the endozoochorous dispersal of exotic plants (Clergeau & Mandon‐Dalger, 2001; Mandon‐Dalger et al., 1999). Although these novel mutualistic interactions are identified, the extent to which they may favour plant invasions and ultimately trigger long‐term changes in the composition of plant communities remains unknown.

**PLATE 1:**
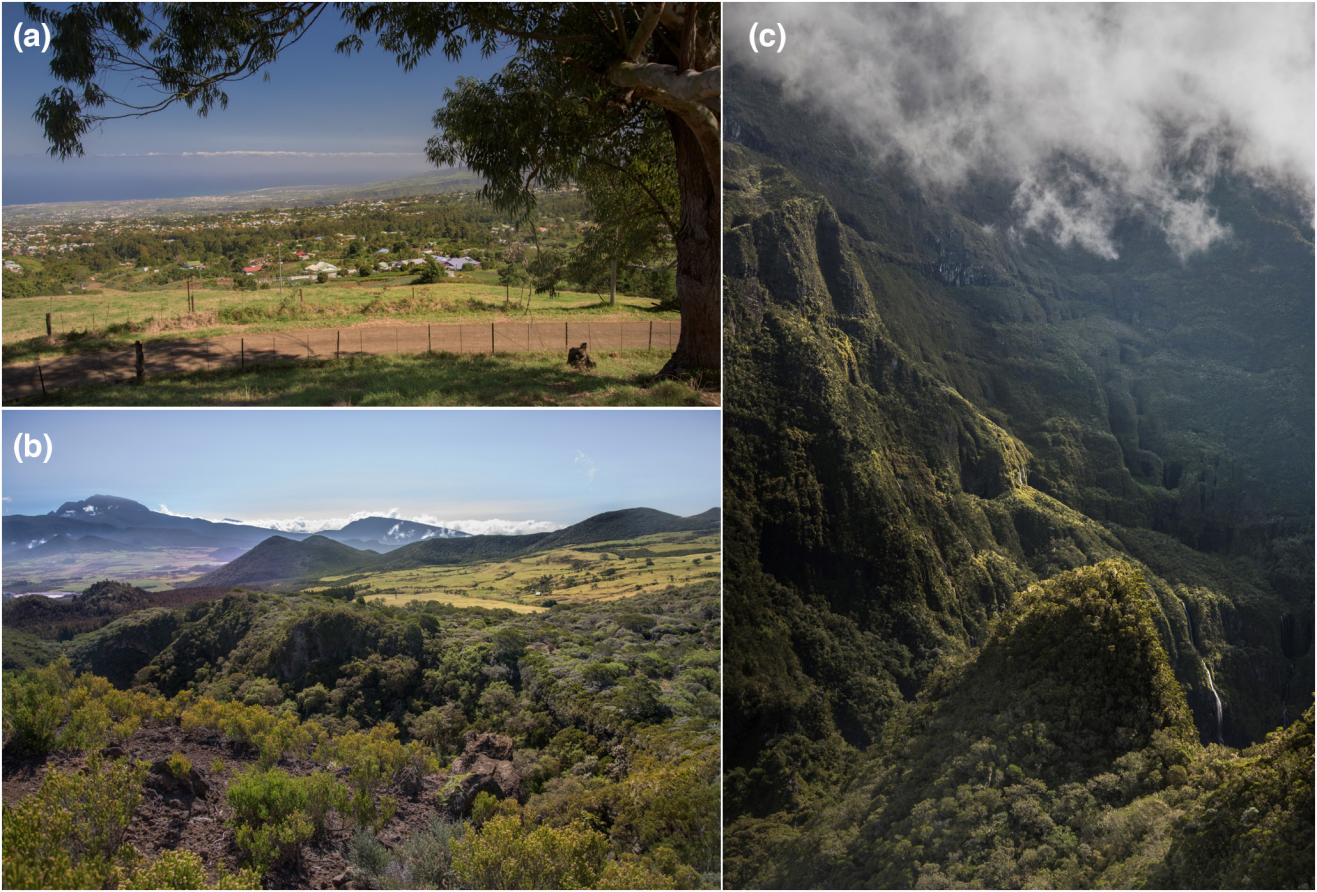
The diversity and spatial structure of habitats in La Réunion island. (a) Background: exotic savannas, and sugar cane crops at low elevation, middle ground: mosaics of exotic thickets, woodlands along cliffs, foreground: mid‐evelation mosaics of pastures and fallows. Sub‐urban and urban habitats are present at all elevation levels. (b) Background: slopes of the Piton des Neiges massif (3070 m asl) covered with high elevation ericoid thickets, mid‐ground: pastures in the central mid‐elevation plain and mosaic of exotic plantations and native forests on higher ancient craters, foreground: native ericoid thickets on the lower slopes of the Piton de la Fournaise massif (2646 m asl). (c) View of a steep high cliff in the centre of the island with native forest of variable stature depending on local slope. All photographic credits (c) Jean‐Yves Barnagaud.

Unlike many oceanic islands with comparable sizes, the land use patterns in La Réunion partly disentangle the distribution of native and converted habitats from altitudinal variation, offering the possibility to distinguish their respective influences on the distribution, composition and diversity of species assemblages (Plate 1). Depending on the scale at which species respond to their environment and resources, the island's fine‐grained mosaics of man‐modified and native vegetation may either inflate contrasts in the functional composition of species assemblages within local habitats, or trigger biotic homogenization at the landscape scale (Byamungu et al., 2021; Cadotte et al., 2011; Diaz & Cabido, 2001; McGill et al., 2006). These features, concentrated on a relatively small study region, make La Réunion a particularly favourable study area to investigate the determinants of spatial variations in the composition of local bird assemblages. In particular, the weak correlation between altitude and human impact, at least up to the upper third of the elevation gradient, allows separating their respective roles on the co‐occurrence or separation of exotic and native bird species among local assemblages.

Building on this specificity, we investigated spatial patterns in the distribution of bird species and their ecological traits in La Réunion, aiming to (i) describe native and exotic species' distributions along habitat and elevational gradients, (ii) investigate the resulting distributions of ecological traits on these gradients and (iii) assess the outcome of these pattenrs in terms of taxonomic and functional diversity patterns. To our knowledge, we thus perform the first comparison of the distributions of native and exotic terrestrial bird species within landscapes on an oceanic island of the Madagascan biome outside Madagascar. From an applied viewpoint, the unpublished data analyzed here were gathered during an extensive bird point count survey conducted in the austral summer 1997–1998, 30 years after the last exotic bird introduction and 10 years before the establishment of La Réunion National Park. Our results therefore provide a useful point of reference for a future evaluation of conservation measures aimed to reduce human imprint of native ecosystems within the national park (McNellie et al., 2020).

## 2 MATERIALS AND METHODS

### 2.1 Study location

**FIGURE 1:**
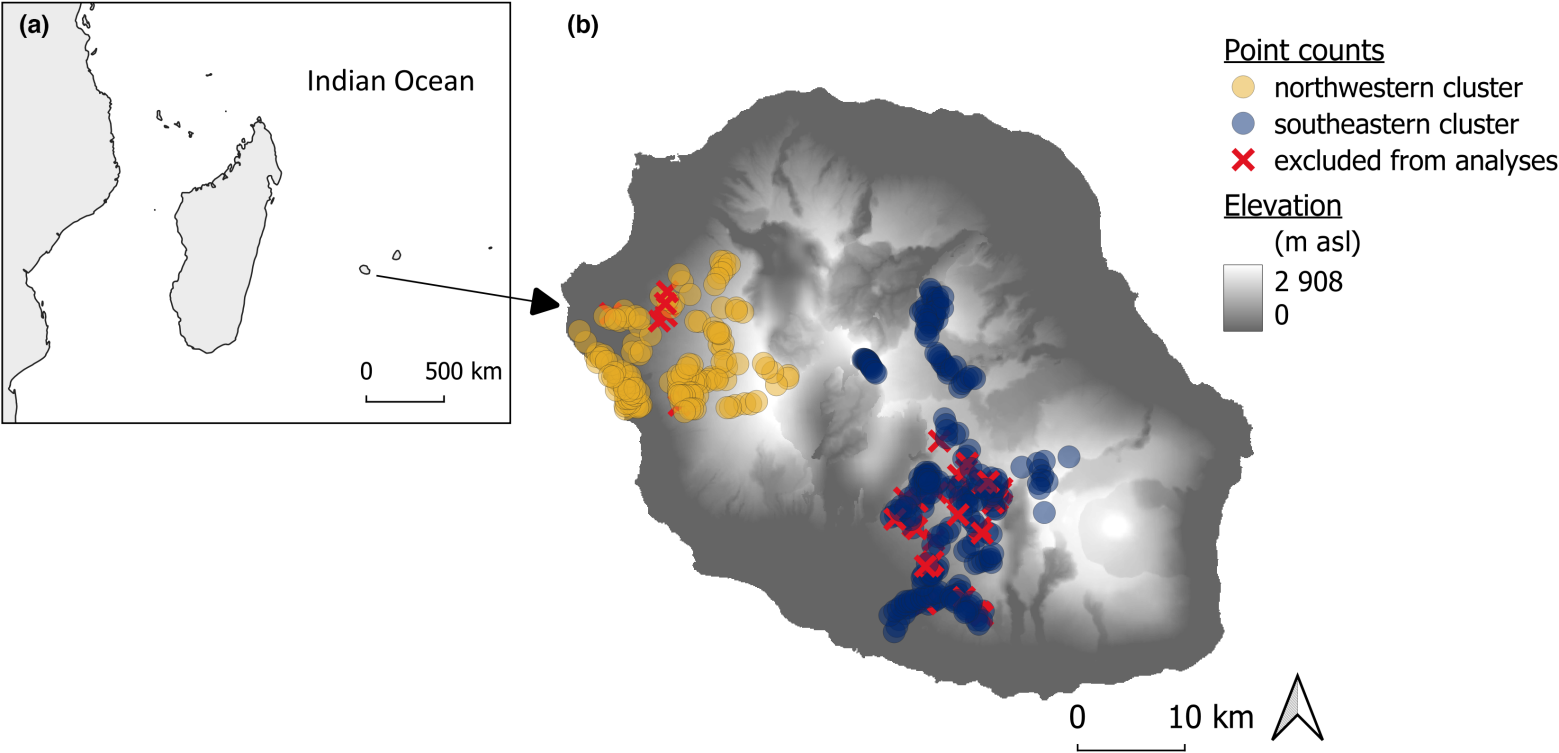
Location of La Réunion island (a) and sampling design (b). The 38 red crosses depict points removed from the analysis due to missing data. Background layer: elevation raster (Jarvis et al., 2008).

La Réunion Island is a 3‐million‐year‐old volcanic island, part of the Mascarene archipelago (Western Indian Ocean, 55°30' E and 21°05' S, Figure 1). Administratively, it is a french ‘département d'Outre‐Mer’, which warrants the feasibility of long‐term studies with the support of well‐established local research institution and conservation stakeholders, a relatively rare feature in the Madagascan region. In the centre of the island, a plateau (Plaine des Cafres, ca. 1500 m above sea level) is flanked by two volcanoes (Piton de la Fournaise, 2631 m, active, and Piton des Neiges, 3069 m, extinct), descending into the coastal plain with an elevation change of 2000 m in 10–30 km. This topography causes a steep climatic gradient, with an average minimum ground temperature below 0°C above 1500 m in August, above 1800 m from June to October, and above 2300 m all year round. At the highest point, the annual temperature variation ranges from −10°C to +26°C (Cadet, 1974; Raunet, 1991). The study area, located on the leeward side of the island, has a slope of about 0.15 m per m and an annual rainfall ranging from 500 mm at sea level to 2000 mm on the highest mountain slopes (Raunet, 1991). The vegetation is organized in elevational belts arranged from sea level to mountain tops. Partly orthogonal to this habitat gradient is a gradient of human habitat conversion. The original ecosystems have been modified earlier and more intensively at low elevations; patches of native tropical mountain forests still remain at mid elevations (NATI in Table 1, the most important remaining native forest type in terms of surface and conservation). High elevations are still mostly uninvaded and covered by endemic vegetation (HIER and ACFO in Table 1, Albert, Flores, Ah‐Peng, & Strasberg, 2021; Cadet, 1980). The main habitat belts sampled in this study can roughly be categorized into coastal urbanized plains (0–50 m), savannah (50–300 m), sugarcane monoculture (300–800 m), rural landscapes with crops (800–1200 m), primary forest mixed with pastures and planted forests (1200–1800 m), heathlands dominated by ericaceous plants (1800–2600 m) and bare soils with sparse herbaceous vegetation (2600–3069 m).

**TABLE 1:** Habitat types used in the study and acronyms.

Label	Variable	Type	Mean ± SD [range]	Elevation barycentre [range] (m)
ELEV	Elevation (m)	Quantitative	1202.3 ± 649.2 [20, 2880]	
ACFA	*Acacia mearnsii‐*dominated fallows	Proportion in 150 m around each sampling point (expressed in %)	12.4 ± 29.2 [0, 100]	1263 [640, 2070]
ACMI	Degraded native forest mixed with *Acacia mearnsii*		0.4 ± 4.2 [0, 70]	1746 [1510, 2880]
NATI	Native mid‐elevation forest («Bois de couleurs»)		12.9 ± 31.9 [0, 100]	1398 [620, 2540]
HIER	High‐elevation ericoid thickets		9.3 ± 28.9 [0, 100]	2377 [1620, 2880]
SUCA	Sugar cane fields		14.7 ± 34.2 [0, 100]	593 [250, 1010]
CRYPT	Plantations of *Cryptomeria japonica* (conifer)		4.9 ± 15.8 [0, 94]	1025 [320, 1680]
CULT	Cultivated land		4.6 ± 20.6 [0, 100]	189 [60, 320]
EXTH	Thicket of exotic species		5.8 ± 13.4 [0, 72]	1239 [350, 2010]
HEFA	Herb‐dominated fallow		0.3 ± 2.7 [0, 39]	1415 [770, 2010]
SHFA	Shrub‐dominated fallow		10.7 ± 25.1 [0, 100]	1120 [320, 1505]
PAST	Pasture		5.9 ± 21.6 [0, 100]	1583 [630, 1960]
SASH	Mixed savanna‐shruland		3.7 ± 18.6 [0, 100]	125 [20, 230]
SAHE	Savannah (herbs)		3.8 ± 19.6 [0, 100]	64 [20, 170]
ACFO	Monodominant forests of *Acacia heterophylla*		9.3 ± 28.4 [0, 100]	1637 [1400, 2560]
URBA	Urbanized area		1.4 ± 5.7 [0, 46]	1118 [320, 1670]

*Note*: Native habitats were mostly composed of NATI, HIER, ACFA and to a lesser degree ACMI. The Elevation column indicates the elevational barycenter of each habitat, weighted proportionally to the surface covered by this habitat on each site. Figures in brackets give the altitudinal range of each habitat.

### 2.2 Bird sampling

A total of 410 point counts were sampled during the breeding season of austral summer from December 1997 to April 1998. All points were visited once by one of two trained ornithologists (GB and JT), between 5.30 AM and 10.30 AM, under favourable weather (no rain or strong wind). On each point, the observer recorded all birds heard or seen in a 150 m radius during 20 min. Flying birds were only recorded if they were obviously related to the point (e.g. an individual hunting within the 150 m radius). Records are expressed in abundance indices following Blondel et al. (1970, 1981): single bird—1, territorial singing male—2 individuals if the female was not observable.

We discarded 38 points due to incomplete or incoherent environmental data measurements, and thus performed our analyses on 372 points, arranged in two geographical clusters for practical reasons (144 on the north‐western cluster and 228 on the south‐western cluster, Figure 1). Sampling points were evenly distributed all along the elevational gradient, with a mean elevational distance of 8 m between two successive elevations and a horizontal distance of at least 250 m between two successive point counts. All major extant vegetation types were covered within 100 m elevational bands, with a mean sampling effort of 12.7 point counts per band. Wetlands and dry areas such as depressions, ridges and mires were avoided, as well as places too close to feeding troughs in grasslands, because they attract granivorous species.

### 2.3 Habitat data

**FIGURE 2:**
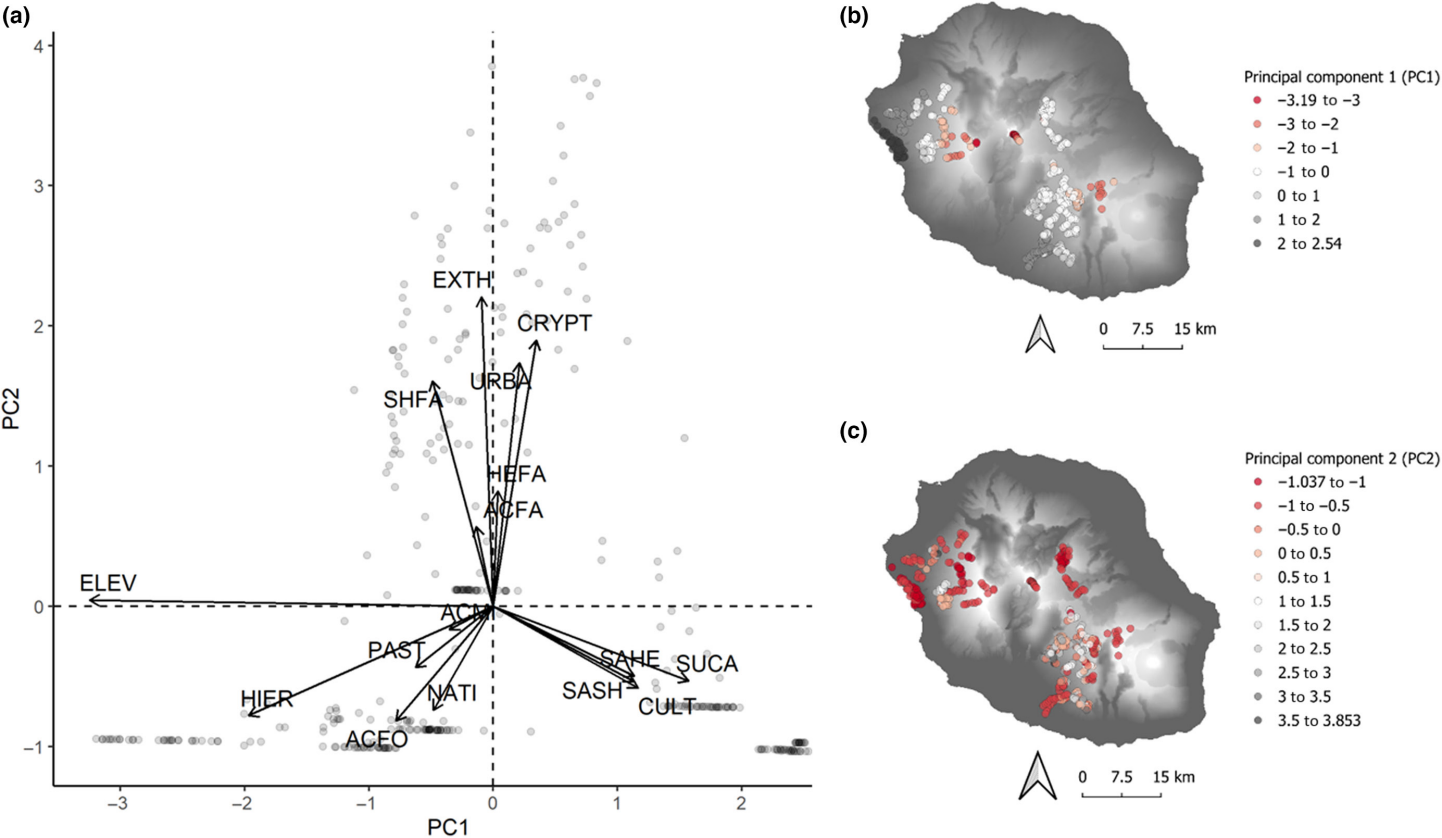
Projection of the 372 bird point counts on the first two axes of a principal component summarizing habitat and elevational variation on the study area (a); maps of principal components values: PC1 (b), PC2 (c). See acronyms for habitat types in Table 1. Background layer: elevation raster (Jarvis et al., 2008).

Landscape composition within a 150 m radius around each point count was characterized as the proportion of the total surface covered by 15 habitat categories (Table 1), quantified through the interpretation of georeferenced aerial photographs acquired in November 1996 at a native 1:5000 scale. We summarized habitat and elevational variation by the two first axes of a principal component analysis (Figure 2, 13% and 10% of total variance for the first and second components, respectively; the third component, which accounted for 8% of total variance, was disregarded). The first principal component (PC1) was dominated by elevation (higher altitudes in negative values) and ranged from lowland cultivations (sugar cane, SUCA and mixed crops, CULT) and savannah (SAHE, SASH) to a variety of mixed exotic and native habitats at mid‐elevations, replaced by endemic *Acacia heterophylla*‐dominated forests (ACFO), and ericoid shrublands (HIER) at the highest elevations. The second principal component (PC2) reflected the spatial heterogeneity of local habitats. It opposed a variety of homogeneous sites including native or exotic habitats (negative values), to sites characterized by mosaic habitats with high within‐sites heterogeneity, mostly dominated by exotic vegetation (positive values): fallows (HEFA, SHFA, ACFA), exotic thickets (EXTH), plantations (CRYPT) and urban habitats (URBA).

### 2.4 Species' status and ecological traits

From the 23 species recorded at least once on the point counts, we discarded three species observed less than three times, since we considered them to be insufficiently represented to assess their habitat affinities (these species are listed in Appendix S1).

We defined species' status based on Safford et al. (2015) as either ‘native’ (nine species endemic to the island or having colonized it without human assistance) or ‘exotic’ (11 species introduced by humans, mostly in the 19th century for ornament or hunting). For these 20 species, we compiled five ecological traits that represent key phenotypical, behavioural and biogeographical characteristics, and may affect their habitat affinities: species' biogeographic origin (exotic species' zoogeographic region of origin, all native species belong to the Madagascan biome), main diet and dietary specialization, main vegetation strata used for foraging and body mass (log‐transformed to limit the influence of a few large values). These traits are classical proxies of species' patterns of habitat selection, home range and resource use. Their distributions among species assemblages are therefore expected to change in response to habitat filtering and competitive exclusion processes, allowing to investigate the role of niche‐related processes in the separation or co‐occurrence of native and exotic species in habitats. Similar traits were associated with the distinct habitat preferences of exotic and native birds in a previous study focused on New Zealand (Barnagaud et al., 2022).

We used species‐level trait averages synthesized from multiple sources and published in the Handbook of the Birds of the World (Del Hoyo et al., 2013, see Appendix S1 for trait values, their computation and references therein). Intraspecific trait variance may be high especially in an insular context, even for recently established species, which may diverge phenotypically from their source populations in a few generations (Wiens et al., 2019). However, trait data at the scale of local populations are currently lacking for most species in La Réunion as in many other oceanic islands. We tempered this limitation by considering a limited number of traits with rather coarse resolutions, thus ensuring that they actually reflect ecological differences among species. Given the low size of our species assemblage, using more traits would result in over‐splitting ecological variability among species, likely yielding uninterpretable patterns.

### 2.5 Statistical analyses

We summarized the variation of these five traits among the 20 species in a Hill & Smith analysis (an ordination method for tables mixing quantitative and qualitative variables, Hill & Smith, 1976) in order to explore the ecological overlaps and separations between native and exotic species. We then explored trait‐habitat associations for the 20 species and 372 points in a three‐table ordination method known as the RLQ analysis (Doledec et al., 1996), which links a matrix of environmental variation across sampling sites (table L, based on the PCA described in the Habitat data section) to an ecological trait matrix (table Q, based on the Hill & Smith analysis described in the Section 2.4), through a sites × species matrix (table R, here a correspondence analysis on species' indices of abundance per point, see Section 2.2).

We visually explored the RLQ space to search for trait–habitat associations that may distinguish native from exotic species, and tested the robustness of trait–habitat associations through two complementary permutation models (Thioulouse et al., 2018, p. 231). The first model tested the association between R and L (observed association vs. 999 permutations under the null hypothesis that species are distributed randomly across sites). The second model tested the association between L and Q (permutations under the null hypothesis that traits are randomly distributed across species assemblages). The robustness of the association between R and Q corresponded to the highest *p*‐value between these two tests. We then tested phylogenetic signals on axes scores of the Hill & Smith analysis and RLQ analysis with Pagel's λ and Blomberg's K, as a sensitivity analysis to assess the role of species' evolutionary relatedness in the observed trait‐environment associations (phylogenetic tree, details on the method and results in Appendix S3).

To investigate variations in assemblage diversity, we computed point‐level species richness, Shannon's index, and functional dispersion (FDis: the average distance of species to the centroid of each assemblage in a multivariate trait space formed by the five ecological traits described above, Laliberté & Legendre, 2010). We discarded other functional diversity metrics to avoid an inflation of statistical tests, especially because most of them are at least partly redundant. In this respect, FDis is well suited to our study since it provides an intuitive and integrative measure of functional diversity compared to the mean functional assemblage (i.e. the spread of species forming a local assemblage in a multivariate trait space) while being computationally uncorrelated to species richness (Laliberté & Legendre, 2010).

We analysed diversity indices separately for three species groups: all species, native species only or exotic species only (maps of the indices in Appendix S2). We used generalized additive models (GAM, Wood, 2006) to express diversity indices as a function of thin plate spline smoothers of the two first principal component axes of the environmental PCA (PC1 and PC2), plus a covariate separating the two geographical clusters of points (locations of the two clusters in Figure 1; estimates of the cluster effect are provided in Appendix S4). We fitted species richness as a Poisson distribution, and Shannon's index and functional dispersion as Gaussian distributions. Residuals of all models were slightly structured in space, but the magnitude of the spatial autocorrelation in the first orders of neighbourhood was so low (Moran's I < 0.05 within a few kilometers) that we did not proceed with further correction.

We performed all analyses under the R 4.1.2 environment (R Core Team, 2021) with packages ade4 (Thioulouse et al., 2018), picante (Kembel et al., 2014) and mgcv (Wood, 2006).

## 3 RESULTS

### 3.1 Trait overlap between native and exotic species

**FIGURE 3:**
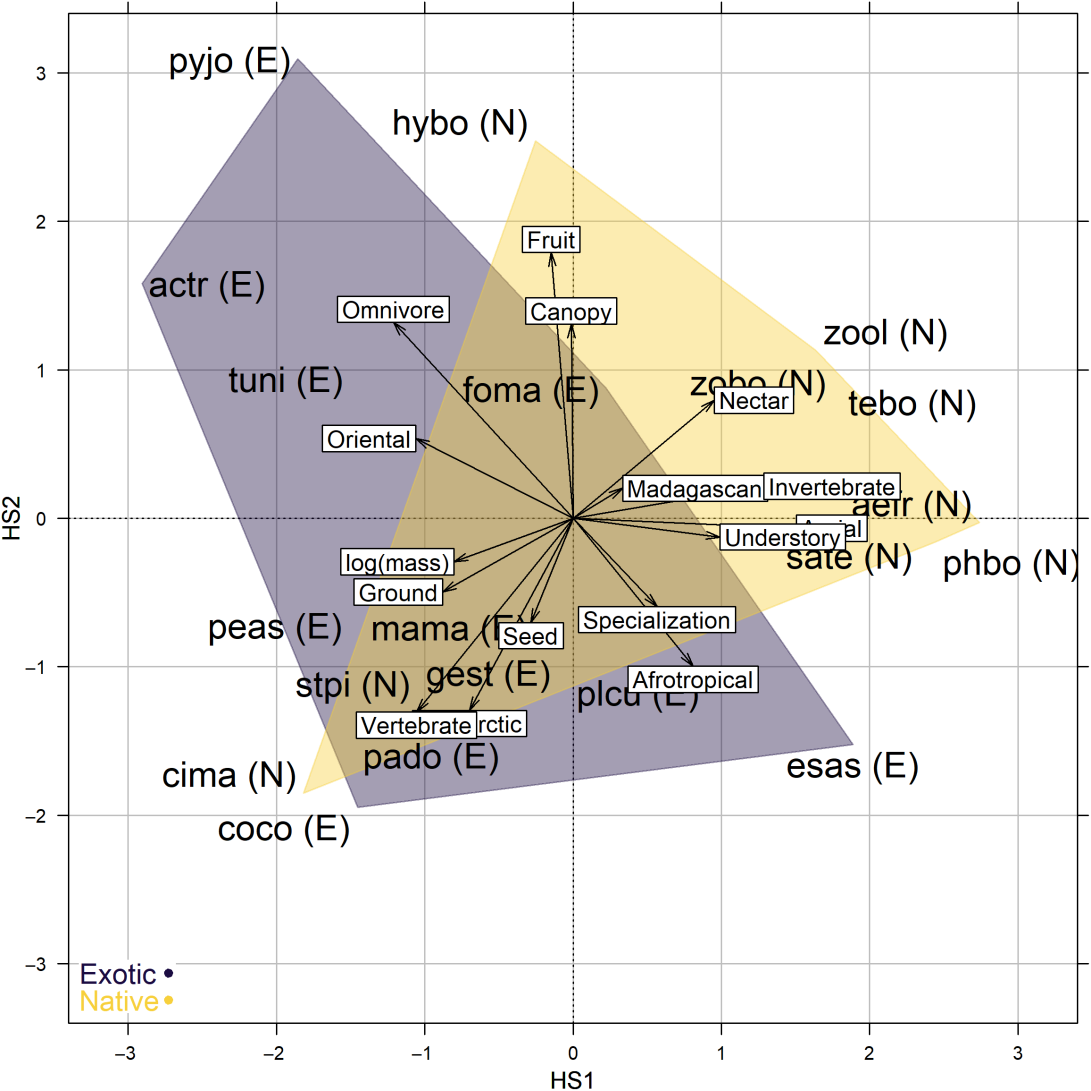
The ecological trait space of 22 bird species based on five ecological traits (biogeographic origin, main diet, dietary specialization, foraging vegetation strata and log‐transformed body mass), with convex hulls separating native (N) and exotic (E) species. Species' acronyms are defined in Appendix S1.

The two first axes of the Hill & Smith analysis summarized 23 and 16% of the total trait variation among the 20 species (third axis 14%, disregarded). The 20 species were evenly dispersed in the multivariate trait space, indicating relatively low ecological redundancy and few extreme strategies with respect to the traits used (Figure 3). The first axis (HS1) separated relatively large, ground‐foraging omnivorous species of oriental origin (e.g. Madagascar Buttonquail—tuni, Common Myna—actr or Red‐whiskered Bulbul—pyjo; refer to Appendix S1 for scientific names) from dietary specialists (mostly native insectivores or nectarivores: Mascarene Swiftlet—aefr, Mascarene Martin—phbo, and Reunion Stonechat—sate, and one ubiquitous exotic granivorous species, Common Waxbill—esas). The second axis (HS2) separated introduced Palearctic and African species with seed‐based regimes (Common Waxbill—esas, Common Quail—coco, Village Weaver—plcu) from frugivores and insectivores, such as forest‐prone native species (Reunion Bulbul—hybo, and Reunion White‐Eye—zool) or exotic forest species (Red‐whiskered Bulbul—pyjo). Most of the overlap between native and exotic species was attributable to two species, Reunion Harrier (cima, eight occurrences in our data), the only extant endemic bird of prey, and Malagasy Turtle Dove (stpi, 38 occurrences in our data). Species scores along HS1 and HS2 did not depart from a Brownian evolution model (tests of phylogenetic signal in Appendix S3).

### 3.2 Native and exotic species' distributions along the elevation and habitat gradients

**FIGURE 4:**
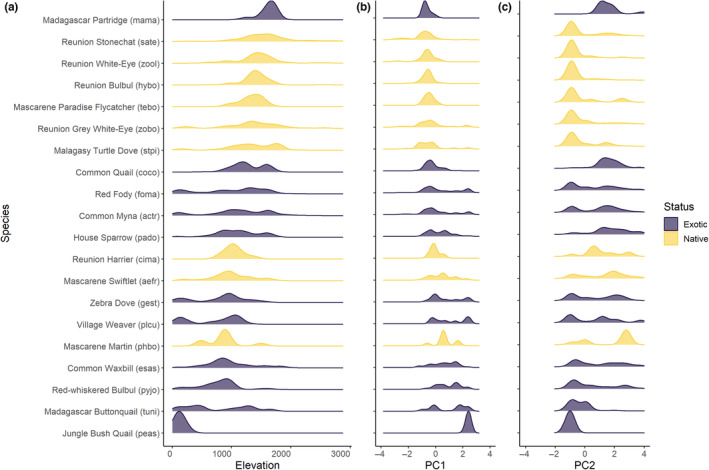
Count density of the 22 native and exotic species recorded in the survey along the elevation gradient (a) and the two first axes of the principal component analysis on habitat variables (b, c) (no density curve for Blue‐breasted Quail *Synoicus chinensis* since it occurred only once in the data set).

Native and exotic species were intertwined along the elevation gradient, but natives tended to predominate at higher altitudes and were virtually absent from coastal sites dominated by exotics (native species' mean elevation = 1395 m, SD = 478 m, range = 60–2880 m, Figure 4a). The Reunion Harrier (cima) and the two aerial feeders (Mascarene Swiflet – aefr, and Mascarene Martin – phbo) were present at lower altitudes than most native passerines. Exotic species had similar elevation ranges, yet their altitudinal distribution was skewed towards coastal plains (exotic species' mean elevation = 962 m, SD = 499 m, range = 20–2400 m, Figure 4b).

Native species tended to avoid the most human‐disturbed and fragmented habitats (higher counts in the negative side of PC2, Figure 4c), with the exceptions of the Reunion Harrier (cima) and the two aerial feeders that showed a larger habitat range on PC2 (Mascarene Martin—phbo, and Mascarene Swiftlet—aefr). Exotic species living in the lowlands (positive PC1 values) also tended to avoid highly fragmented and urbanized areas (i.e. exotics located in positive PC1 values were more abundant in negative PC2 values).

### 3.3 Trait–environment associations

**FIGURE 5:**
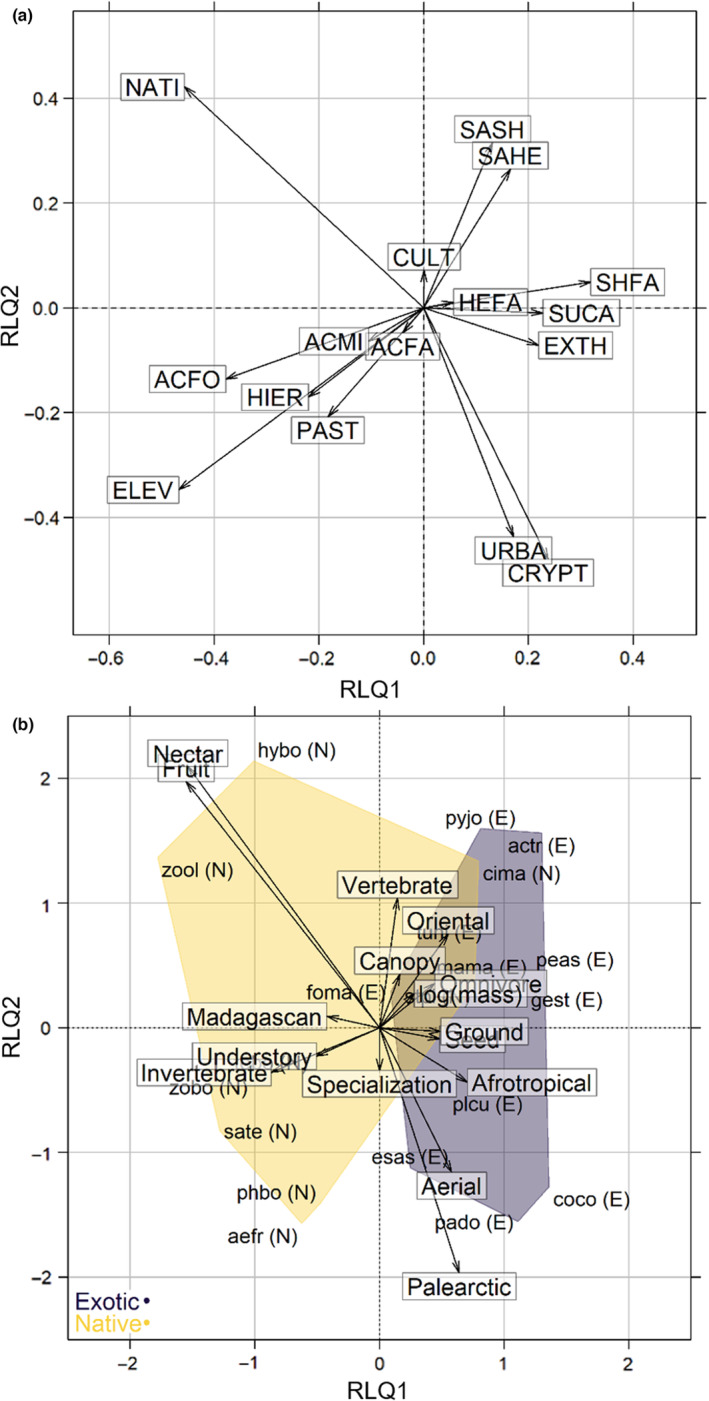
Trait‐environment associations in a RLQ space (projection of habitat variables in (a), traits in (b), with exotic and native species' convex hulls). See acronyms for habitat types in Table 1.

Species‐habitat relationships were robust to permutations of tables R and L (‘Model 2’ in Thioulouse et al., 2018, observed sum of eigenvalues = 1.6, *p* = .001 after 999 permutations), but not to permutations of Q and L (‘Model 4’ in Thioulouse et al., 2018, observed sum of eigenvalues = 1.6, *p* = .07 after 999 permutations), indicating that ecological traits were overall not strongly segregated along habitat gradients. This lack of power, in spite of clear‐cut patterns in Figure 5, may well have resulted from the relatively low species and trait sample sizes, as from a low trait turnover along ecological gradients. For instance, in the RLQ space, fruit‐ and nectar‐eaters were strongly related with native mid‐elevation forests (NATI, Figure 5), but these two diets were represented by only one native species each (Reunion Bulbul—hybo, and Reunion Olive White‐eye—zool, left top corner of the RLQ space). On the opposite side of the RLQ space, the only two palearctic species introduced on the island (Common Quail—coco, and House Sparrow—pado) and, at higher elevations, aerial feeders (Mascarene Swiftlet—aefr, and Mascarene Martin—phbo), were associated with urbanized areas and *Cryptomeria* plantations (negative coordinates along RLQ2, Figure 5). As for trait multivariate axes, the distribution of species along RLQ1 and RLQ2 did not depart from a Brownian evolution model (Appendix S3).

### 3.4 Diversity–environment associations

**FIGURE 6:**
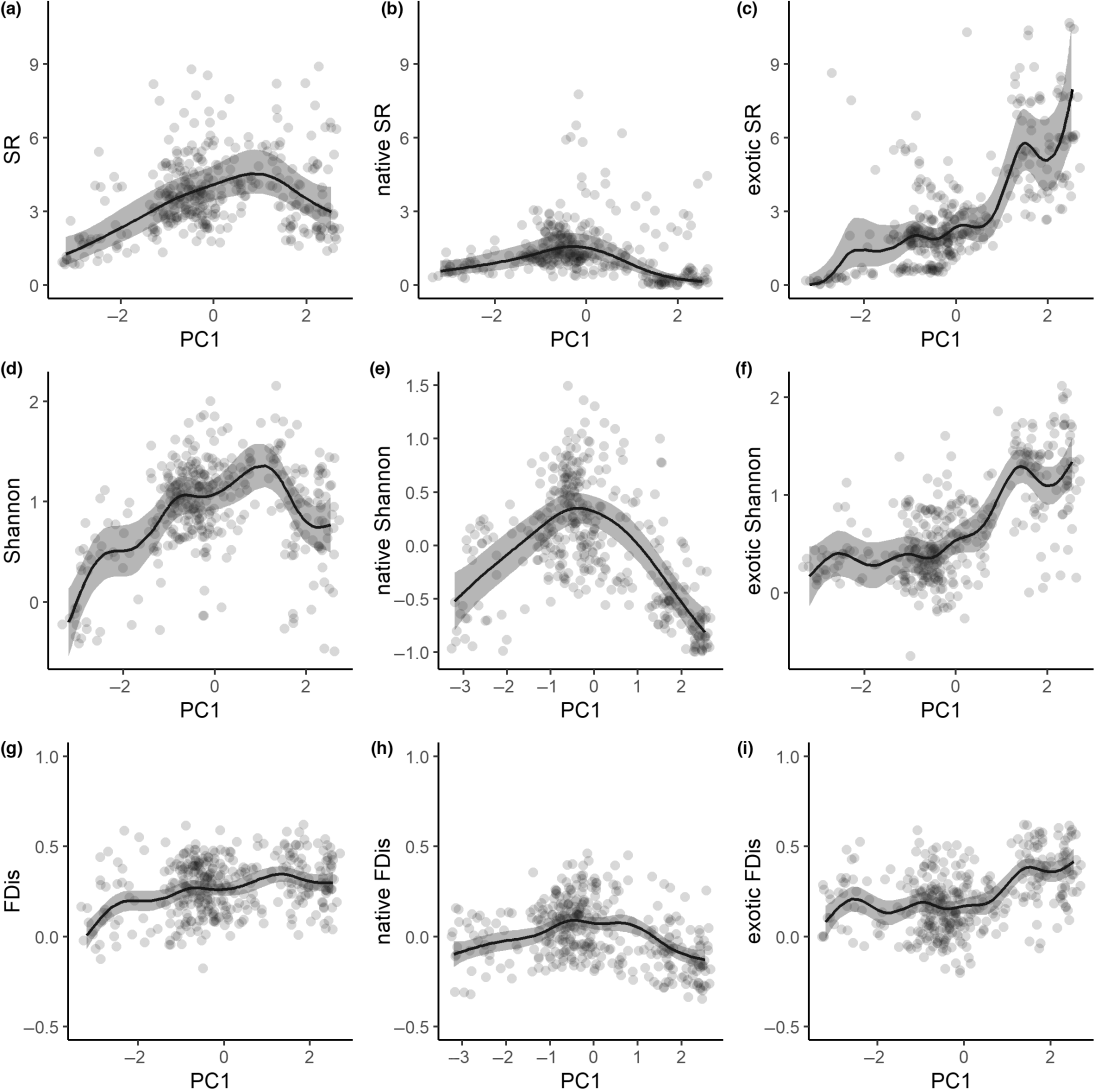
Variation of species richness (SR), Shannon's index (Shannon) and functional dispersion (FDis) along a principal component axis synthesizing an elevational gradient associated with spatial turnover in vegetation types (PC1, native vegetation at higher elevations in negative values, exotic vegetation in lowlands in positive values). From left to right: all species, native species, exotic species.

**FIGURE 7:**
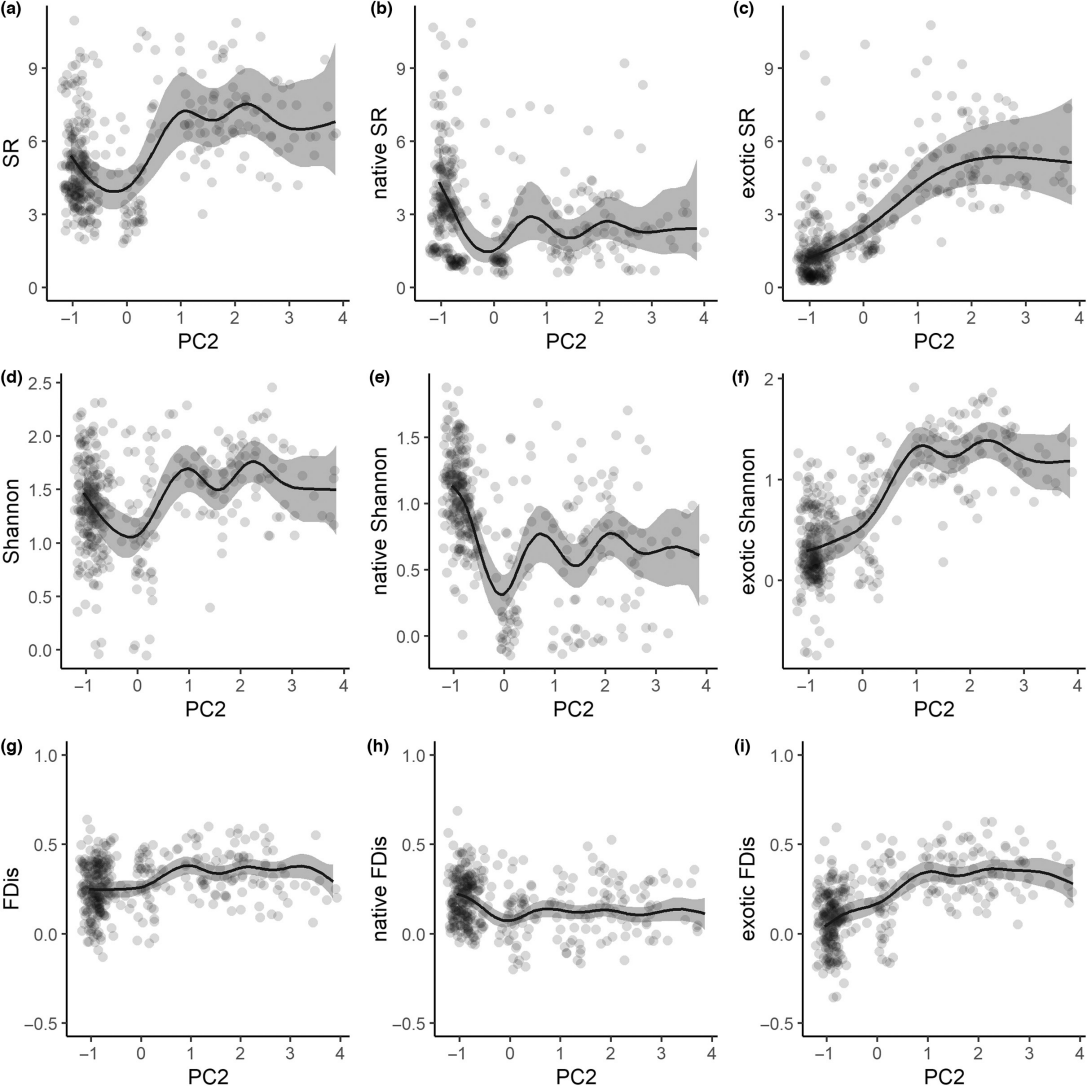
Variation of species richness (SR), Shannon's index (Shannon) and functional dispersion (FDis) along a principal component axis synthesizing habitat heterogeneity within sampling sites (PC2, homogeneous sites in negative values, heteregeneous sites showing mosaics of native and anthropogenic habitats in positive values). From left to right: all species, native species, exotic species.

The GAMs showed that species richness, Shannon's index and functional dispersion varied non‐linearly along PC1 (Figure 6) and to a lesser extent along PC2 (Figure 7). Species richness and Shannon's index exhibited hump‐shaped relationships with PC1, with a maximum slightly shifted towards low‐elevation areas (Figure 6a,d, positive scores), mainly due to the near‐absence of native species in coastal plains (Figure 6b). Inversely, exotic species richness and Shannon's index decreased steadily from lower to higher elevations (Figure 6c,f, i.e. increased along PC1). Total and exotic species' functional dispersion decreased slightly from lower to higher elevations (Figure 6g,i), but peaked at mid‐elevations for native species (Figure 6h).

Total species richness was depleted, and Shannon's index was slightly lower, in homogeneous sites with either native or exotic habitats (negative PC2 values, Figure 7a,d), in contrast to heterogeneous sites composed of interspersed exotic habitats and urban areas (positive PC2 values). Exotic species richness and Shannon's index peaked at intermediate levels of PC2 before plateauing (Figure 7c,f). Inversely, the highest native species richness and diversity occurred in homogeneous native habitats, then declined slightly to a lower plateau with high variation (Figure 7b,e). FDis varied little along PC2 overall (Figure 7g). However, native species' FDis was slightly higher in homogeneous sites with native habitats (negative PC2 values in Figure 7h). Conversely, it increased slowly towards urban areas and exotic thickets up to a plateau for exotic species (positive PC2 values in Figure 7i).

## 4 DISCUSSION

Most native species were tied to mid‐and high‐altitude habitats dominated by indigenous vegetation, while the abundance distributions of exotic species spread along a wider gradient of habitats and elevations. Although insular avifaunas tend to be composed of ecologically flexible species (Prodon et al., 2002; Reeve et al., 2018), native species clearly displayed narrow niche width along the two gradients studied, contrasting with the flatter abundance distributions of many exotic species. The discrepancy between species' habitat distributions among the two guilds was therefore not as obvious as expected from other comparable studies on other oceanic islands (Acevedo & Restrepo, 2008; Barnagaud et al., 2014; Flaspohler et al., 2010; Hahn et al., 2011; van Heezik et al., 2008).

**PLATE 2:**
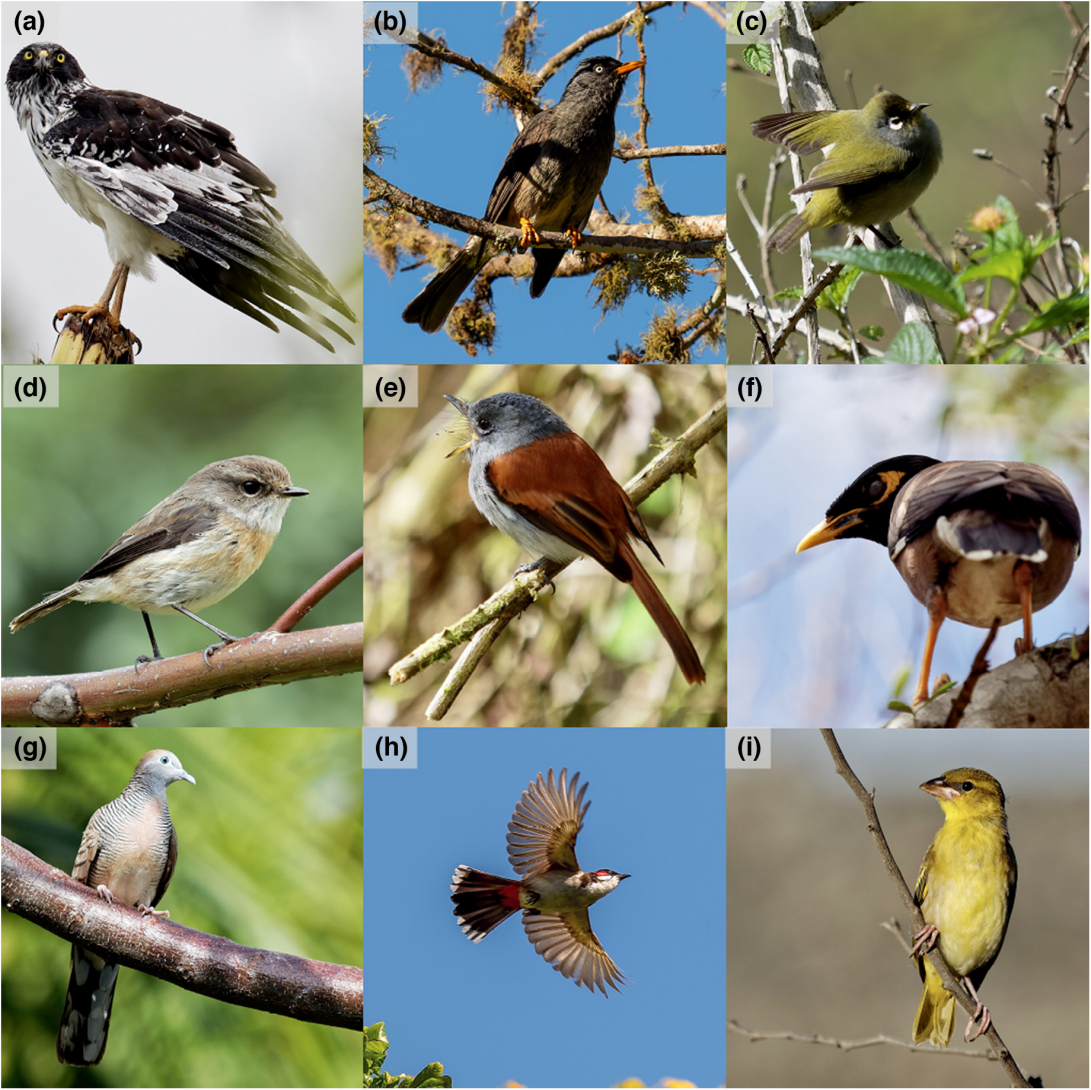
Some common native and exotic species represented in our data: (a) Reunion Harrier (cima, native), (b) Reunion Bulbul (hybo, native), (c) Reunion White‐Eye (zool, native), (d) Reunion Stonechat (sate, native), (e) Mascarene Paradise Flycatcher (tebo, native), (f) Common Myna (actr, exotic), (g) Zebra Dove (gest, exotic), (h) Red‐whiskered Bulbul (pyjo, exotic), (i) Village Weaver (plcu, exotic). All photographic credits (c) Paul‐Alexandre Leclerc.

In spite of these abundance patterns, field observations show that native species in La Réunion frequent a relatively wide range of native habitats (e.g. Reunion Stonechat or Reunion White‐eye, which occur from mid‐elevation forests to open scrubs at mountain tops) (Plate 2), while the vast majority of exotic species do not divert from anthropogenic settlements and exotic habitats for more than a few 100 m, irrespective of altitude (authors' *personal observations*). These observations conform to patterns observed on other islands (Acevedo & Restrepo, 2008; Barbaro et al., 2012; Barnagaud et al., 2014; Hahn et al., 2011; Thibault et al., 2018; van Heezik et al., 2008). Narrower abundance distributions in native species thus likely reflect the reduced availability of native habitat and resources within landscapes, while the development of agriculture on mid‐altitude plateaus have provided exotic species adequate conditions for their expansion along the elevation gradient.

Converging with this niche‐based interpretation, native and exotic species showed distinct ecological trait syndromes and were well separated in the RLQ space. We did not detect any phylogenetic signal contributing to shape these trait‐environment relationships, which may be due to lack of statistical power, but more likely suggests that native and exotic species' distributions along the island environmental gradients do not stem from niche conservatism alone. This pattern aligns with results obtained in New Zealand, where exotic and native species also bear distinct trait syndromes and habitat preferences at a landscape scale (Barnagaud et al., 2022). In the case of La Réunion, trait–environment relationships were predominantly explained by the distribution of species along environmental gradients rather by than traits themselves, suggesting that habitat barriers are independent from the traits we considered.

Still, we found that most frugivorous, insectivorous and nectarivorous native species were associated with high elevation and native forests, and did not come in contact with ground‐dwelling omnivorous and granivorous exotic species, which are limited to man‐modified habitats. Irrespective to whether or not ecological traits trigger an ecological filter, these results conform to the hypothesis that exotic species opportunistically remained confined to their historical habitats of introduction (Blackburn et al., 2013; Duncan et al., 2003), even though successfully introduced species usually exhibit traits enabling them to cope with novel environments (Blackburn et al., 2009). Conversely, native species may not be excluded from modified landscapes because their ecological traits prevent them from using man‐related resources, but more likely due to other processes such as predation, competitive exclusion by exotic species, fine‐grained ecological specialization, or preference for large extents of undisturbed habitats (Parlato et al., 2015).

Native aerial feeders such as Reunion Harrier, Mascarene Swiflet and Mascarene Martin had wider abundance distributions along the axis of habitat heterogeneity and were closer to exotic species than other native species in the RLQ space. These species cover large distances for foraging and thus depend less on the composition of local habitats where they were detected. Conversely, the frugivorous Red‐Whiskered Bulbul was closer to native forest species than most other exotic species in the RLQ space. This recently‐introduced bulbul is currently restricted to ecotone areas and infrequently enters native forests, but it has a high propensity to fast colonization after introduction and naturalization (Clergeau & Mandon‐Dalger, 2001), enabling it to rapidly adapt to novel environments (Amiot et al., 2007). It is also well known for spreading invasive exotic plant species through zoochory (Mandon‐Dalger et al., 1999). Hence, its possible expansion into uninvaded habitats needs to be cautiously monitored since it may pose serious conservation issues for native plant assemblages, which can moreover be affected by a reduction of their dispersal by local functional extinctions of native birds (Anderson et al., 2021).

Diversity patterns were in line with species‐level analyses. Native species reveal hump‐shaped curves of diversity along the mixed elevation/habitat type gradient (PC1), reflecting the higher availability of native habitats at mid‐elevations. Meanwhile, monotonous variation in all forms of exotic species' diversity was coherent with their reluctance to enter native habitats and generally to disperse out of human settlements (Duncan et al., 2003; Sol et al., 2017). Interestingly, the role of habitat heterogeneity (PC2) was low for native species, although they were more diverse in non‐fragmented native habitats. By contrast, and as expected, exotic species were clearly more diverse in habitat mosaics, corresponding to former forest areas converted to agriculture and urban settlements. Functional dispersion also matched these patterns, suggesting that traits are sampled in local assemblages according to species richness rather than under the influence of strong ecological filters, in coherence with the permutation tests performed on the RLQ analysis.

These habitat‐related patterns of assemblage composition may change in future due to continuing anthropogenic pressure on habitats, selective habitat restoration and species protection or control. At a landscape scale, modifications of interaction networks and the emergence of new eco‐evolutionary processes may arise if individuals of trophically‐similar species come increasingly into direct contact in the future (Lugo et al., 2012). Given our results, this threat is however unlikely in the current avifauna of La Réunion, unless recently‐introduced species, such as the frugivorous Ring‐necked Parakeet Psittacula krameri in 1972 (currently subjected to eradication campaigns, Caceres et al., 2022) or the Red‐billed Leiothrix (Leiothrix lutea, Tassin & Rivière, 2001), manage to establish feral populations in native habitats. Once established, these novel colonizers might unpredictably create novel species–resource interactions through e.g. zoochorous seed dispersal (Anderson et al., 2021), as is currently the case for the red‐whiskered bulbul in La Réunion. Furthermore, elevational shifts in bird ranges could ultimately cause thermal squeezes in response to climate change (Freeman et al., 2018; Walker et al., 2019). However, historical records on species distributions are still insufficient to document such long‐term dynamics and the resulting assemblage‐level modifications that would reflect potential thermal squeezes. In the case of La Réunion, our study was based on the oldest protocoled survey of bird communities with sufficient sample size and spatial coverage, making it a potential point of reference for future diachronic studies focused on the effects of habitat or climate changes (McNellie et al., 2020).

## 5 CONCLUSION

As on other islands, the avifauna of La Réunion is not a single ‘novel’ species assemblage (Lugo et al., 2012), but consists of two distinct species assemblages separated by local habitat, which merge together at the landscape scale mostly around mid‐elevations where sufficient amounts of native habitats persist within an otherwise man‐modified matrix. Apart from the specific case of the Red‐whiskered Bulbul, which could still be undergoing a positive colonization dynamic, recent field observations did not suggest substantial changes in this segregation pattern. However, La Réunion habitats and landscapes have changed in the 25 years since our data collection, due to the conflicting effects of increasing anthropogenic pressures and efforts to preserve wide expands of native habitats in the National Park. Replicating our field protocol may allow a quantitative, landscape‐level overview of the consequences of these changes for the composition of bird assemblages. Given the high pace of ecosystem‐level changes experienced on oceanic islands and its consequences for endemic insular biodiversity, we suggest that long‐term studies and surveys become a priority for local conservationists and stakeholders. We also advocate in favor of further analysis of historical data sets on other islands, including still unpublished data wherever they exist, to increase the availability of reference points enabling the evaluation of conservation‐oriented policies in protected areas, especially when established on insular ecosystems.

## AUTHOR CONTRIBUTIONS

**Jean‐Yves Barnagaud:** Conceptualization (lead); data curation (supporting); formal analysis (lead); investigation (lead); methodology (lead); supervision (lead); validation (lead); visualization (lead); writing – original draft (lead); writing – review and editing (lead). **Olivier Flores:** Conceptualization (lead); data curation (lead); formal analysis (equal); investigation (lead); methodology (equal); supervision (lead); validation (lead); visualization (lead); writing – original draft (lead); writing – review and editing (lead). **Gérard Balent:** Conceptualization (lead); data curation (lead); formal analysis (supporting); funding acquisition (lead); investigation (equal); methodology (equal); project administration (lead); supervision (equal); validation (supporting); visualization (supporting); writing – original draft (supporting); writing – review and editing (supporting). **Jacques Tassin:** Conceptualization (lead); data curation (lead); formal analysis (supporting); funding acquisition (lead); investigation (equal); methodology (equal); project administration (lead); supervision (equal); validation (supporting); visualization (supporting); writing – original draft (supporting); writing – review and editing (supporting). **Luc Barbaro:** Conceptualization (lead); formal analysis (equal); investigation (equal); methodology (lead); supervision (lead); validation (lead); visualization (equal); writing – original draft (lead); writing – review and editing (lead).

## CONFLICT OF INTEREST STATEMENT

The authors declare no competing interests.

## Supporting information


Appendix S1–S4

## Data Availability

The data and R scripts related to this study are archived on Recherche Data Gouv (https://entrepot.recherche.data.gouv.fr/): doi: 10.57745/BFEXCE.
