# Supplementary material for: Trait‐independent habitat associations explain low co‐occurrence in native and exotic birds on a tropical volcanic island
Source: Ecol Evol. 2023 Jul 20;13(7):e10322. doi: 10.1002/ece3.10322 (PMC10361348; doi:10.1002/ece3.10322)
Supplement: Supplementary file 1 — Appendix S1–S4 [file ECE3-13-e10322-s001.docx]

**SUPPLEMENTARY MATERIALS**

APPENDIX S1 : Species list and ecological traits

APPENDIX S2 : Maps of bird diversity

APPENDIX S3 : phylogenetic tree and tests of phylogenetic signal

APPENDIX S4 : Complements to models

**APPENDIX S1 : Species list and ecological traits**

| **Acronym** | **Species** | **Common names** | **Status** | **Biogeographic**  **origin** | **Main diet** | **Foraging vegetation**  **strata** | **Body mass (g)** | **Dietary specialization** |
| --- | --- | --- | --- | --- | --- | --- | --- | --- |
| actr | *Acridotheres tristis* | Common Myna | Exotic | Oriental | Omnivore | ground | 117.9 | 1.581 |
| aefr | *Aerodramus francicus* | Mascarene Swiftlet | Native | Madagascan | Invertebrate | aerial | 9.7 | 3.536 |
| cima | *Circus maillardi* | Reunion Harrier | Native | Madagascan | Vertebrate | ground | 638.5 | 3.151 |
| coco | *Coturnix coturnix* | Common Quail | Exotic | Palearctic | Seed | ground | 104.4 | 2.816 |
| esas | *Estrilda astrild* | Common Waxbill | Exotic | Afrotropical | Seed | understory | 7.8 | 3.536 |
| foma | *Foudia madagascariensis* | Red Fody | Exotic | Madagascan | Seed | canopy | 16.3 | 2.550 |
| frpo | *Francolinus pondicerianus*  ** removed from analyses (only 2 occurrences)* | Grey Francolin | Exotic | Oriental | Omnivore | ground | 291.4 | 1.389 |
| gest | *Geopelia striata* | Zebra Dove | Exotic | Oriental | Seed | ground | 55.8 | 3.536 |
| hybo | *Hypsipetes borbonicus* | Reunion Bulbul | Native | Madagascan | Fruit | canopy | 54.4 | 2.375 |
| lopu | *Hypsipetes borbonicus*  ** removed from analyses (only one occurrence)* | Scaly-breasted Munia | Exotic | Oriental | Seed | understory | 14.2 | 2.816 |
| mama | *Margaroperdix madagarensis* | Madagascar Partridge | Exotic | Madagascan | Seed | ground | 235.0 | 2.375 |
| pado | *Passer domesticus* | House Sparrow | Exotic | Palearctic | Seed | ground | 28.6 | 2.816 |
| peas | *Perdicula asiatica* | Jungle Bush Quail | Exotic | Oriental | Seed | ground | 69.5 | 2.816 |
| phbo | *Phedina borbonica* | Mascarene Martin | Native | Madagascan | Invertebrate | aerial | 21.0 | 3.536 |
| plcu | *Ploceus cucullatus* | Village Weaver | Exotic | Afrotropical | Seed | understory | 37.7 | 2.816 |
| pyjo | *Pycnonotus jocosus* | Red-whiskered Bulbul | Exotic | Oriental | Omnivore | canopy | 28.3 | 1.581 |
| sate | *Saxicola tectes* | Reunion Stonechat | Native | Madagascan | Invertebrate | understory | 12.7 | 3.536 |
| stpi | *Nesoenas picturatus* | Malagasy Turtle Dove | Native | Madagascan | Seed | ground | 173.5 | 2.816 |
| sych | *Synoicus chinensis*  ** removed from analyses (only one occurrence)* | Blue-breasted Quail | Exotic | Oriental | Omnivore | ground | 39.6 | 1.753 |
| tebo | *Terpsiphone bourbonnensis* | Mascarene Paradise Flycatcher | Native | Madagascan | Invertebrate | canopy | 11.1 | 3.536 |
| tuni | *Turnix nigricollis* | Madagascar Buttonquail | Exotic | Madagascan | Omnivore | ground | 68.8 | 2.315 |
| zobo | *Zosterops borbonicus* | Reunion Grey White-Eye | Native | Madagascan | Invertebrate | understory | 8.1 | 2.121 |
| zool | *Zosterops olivaceus* | Reunion White-Eye | Native | Madagascan | Nectar | understory | 9.4 | 2.375 |

***Sources:***  *Status, biogeographic origin : Safford et al., 2015 ; Main diet and dietary specialization : Barnagaud et al., 2019 (main diet based on the maximum score over 10 dietary items scored from 0 to 10 ; specialization is the standard deviation of these scores ; raw scores from Del Hoyo et al. 2013, available upon request ; not displayed by the data owner’s request); Foraging vegetation strata and body mass from Del Hoyo et al. 2013.*

*Barnagaud, J.-Y., Mazet, N., Munoz, F., Grenié, M., Denelle, P., Sobral, M., Kissling, W. D., Şekercioğlu, Ç. H., & Violle, C. (2019). Functional biogeography of dietary strategies in birds. Global Ecology and Biogeography, 28(7), 1004–1017. https://doi.org/10.1111/geb.12910*

*Del Hoyo, J., Elliott, A., & Sargatal, J. (2013). Handbook of the birds of the world. (Lynx Edicions, Vol. 1–16).*

*Safford, R., Skerrett, A., & Hawkins, F. (2015). Birds of Madagascar and the Indian Ocean Islands (1er édition). Helm.*

**APPENDIX S2 : Maps of bird diversity**


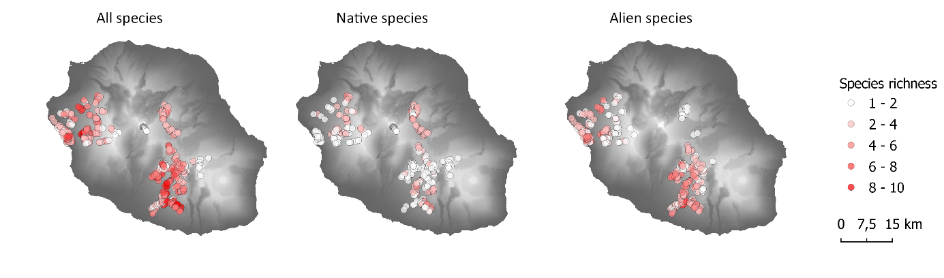


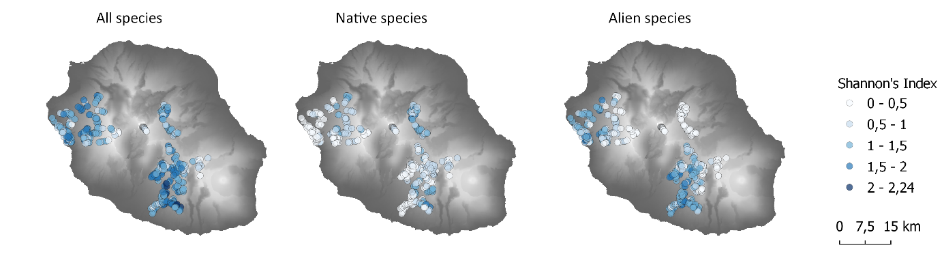


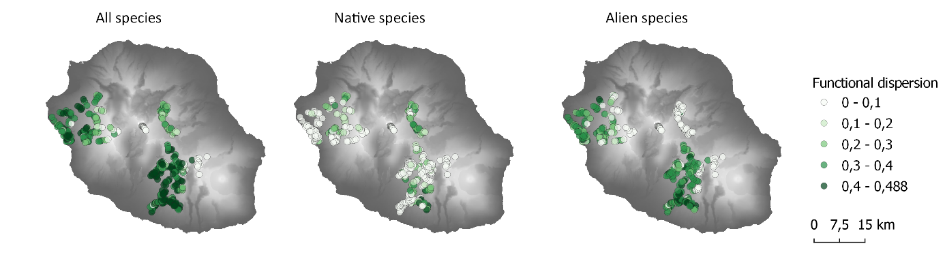


*See main text for the traits underlying functional dispersion. Total species pool : eight native species, 14 exotic species (22 species).Background layer : elevation raster (Jarvis et al., 2008).*

**APPENDIX S3 : Phylogenetic tree and tests of phylogenetic signal**

**Methods**

We constructed a phylogeny of the 20 species with a composite of bird phylogeny established by Prum et al. (2015) and a maximum clade credibility tree computed from 10,000 iterations of the Hackett backbone (Jetz et al., 2012, downloadable at www.birdt ree.org, “Stage 2 Hackett Backbone”), following the method of Cooney et al. (2017) also used in Barnagaud et al. (2022). We then tested for phylogenetic signal on species’ scores on the axes of the Hill & Smith and RLQ analyses using Pagel’s λ and Blomberg’s K, assuming a Brownian evolution model (Phylocom R package, Revell 2012).

**Results**

Table S3.1. Phylogenetic signals on the axes of the Hill & Smith analysis (HS1, HS2) and RLQ analysis (RLQ1, RLQ2).

|  | **Pagel’s λ** | | | **Blomberg’s K** | |
| --- | --- | --- | --- | --- | --- |
| *Variable* | *λ* | *LR(λ = 0)* | *p-value* | *K* | *p-value* |
| HS1 | 0.28 | 0.35 | 0.55 | 0.68 | 0.03 |
| HS2 | 0.64 | 1.84 | 0.17 | 0.62 | 0.04 |
| RLQ1 | 0.97 | 3.73 | 0.05 | 0.71 | 0.01 |
| RLQ2 | 6.77x10^-5^ | -0.0006 | 1 | 0.23 | 0.07 |

*Phylogenetic indices are computed with the function* phylosig *in the phylocom R package (Revell 2012)*. *We assumed a Brownian motion evolutionary model. LR = likelihood ratio test. The p-value associated with Blomberg’s K is based on 1000 permutations.*


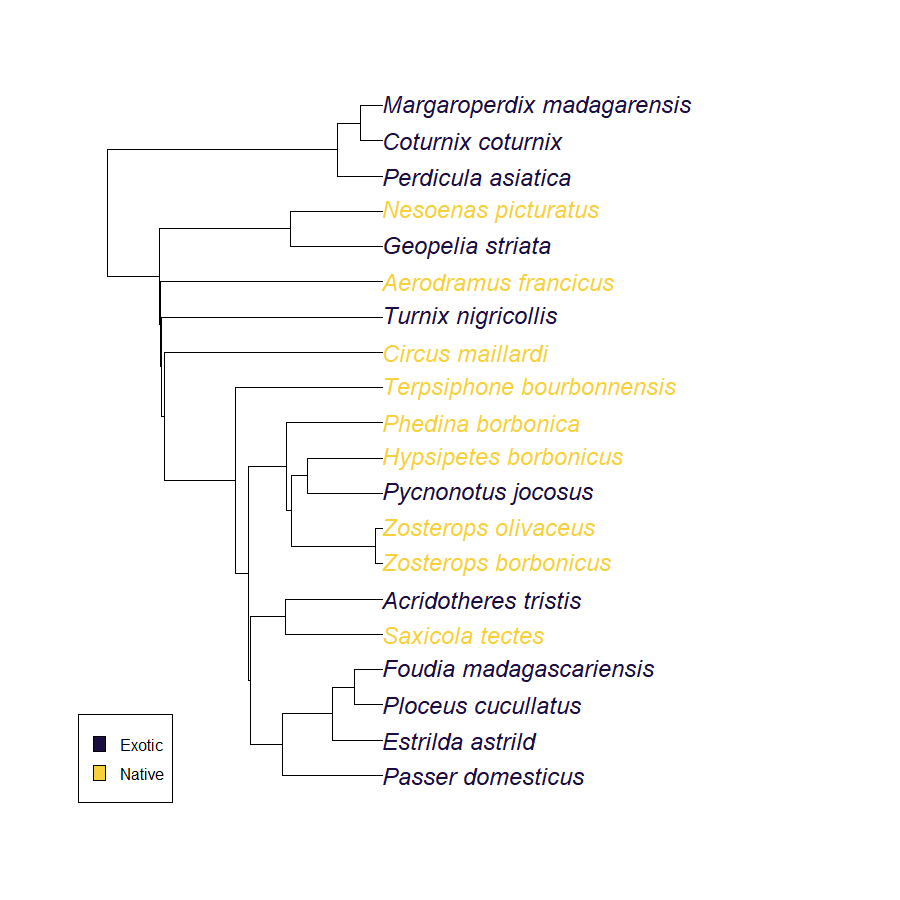


Figure S3.1. Phylogenetic tree of the 20 species.

**References**

Barnagaud, J.-Y., Brockerhoff, E. G., Mossion, R., Dufour, P., Pavoine, S., Deconchat, M., & Barbaro, L. (2022). Trait-habitat associations explain novel bird assemblages mixing native and alien species across New Zealand landscapes. Diversity and Distributions, 28(1), 38–52. <https://doi.org/10.1111/ddi.13432>

Jetz, W., Thomas, G. H., Joy, J. B., Hartmann, K., & Mooers, A. O. (2012). The global diversity of birds in space and time. Nature, 491(7424), Article 7424. <https://doi.org/10.1038/nature11631>

Prum, R. O., Berv, J. S., Dornburg, A., Field, D. J., Townsend, J. P., Lemmon, E. M., & Lemmon, A. R. (2015). A comprehensive phylogeny of birds (Aves) using targeted next-generation DNA sequencing. Nature, 526(7574), 569–573. <https://doi.org/10.1038/nature15697>

Revell, L. J. (2012). phytools: An R package for phylogenetic comparative biology (and other things). Methods in Ecology and Evolution, 3(2), 217–223. https://doi.org/10.1111/j.2041-210X.2011.00169.x

**APPENDIX S4 : Complements to generalized additive models**

Table S4.1. Estimated differences in bird diversity between the two geographical clusters (see Fig.1 in the main text for the locations and spread of the two clusters). The table displays estimated marginal species richness for the two clusters with 95% confidence intervals for the three response variables (SR = species richness, H’ = Shannon’s Index, FDis = functional dispersion) and the three species guilds. Model details are provided in the main text.

|  |  |  | **North-Western cluster** | | |  | **South-Eastern cluster** | | |
| --- | --- | --- | --- | --- | --- | --- | --- | --- | --- |
| ***Response*** | ***Guild*** |  | ***Estimate*** | ***lower bound*** | ***upper bound*** |  | ***Estimate*** | ***lower bound*** | ***upper bound*** |
| SR | All species |  | 4.09 | 3.44 | 4.87 |  | 3.64 | 3.1 | 4.28 |
| SR | Native species |  | 1.52 | 1.13 | 2.05 |  | 1.32 | 0.99 | 1.76 |
| SR | Exotic species |  | 2.36 | 1.9 | 2.95 |  | 2.07 | 1.69 | 2.54 |
| H’ | All species |  | 1.08 | 0.92 | 1.23 |  | 0.97 | 0.82 | 1.12 |
| H’ | Native species |  | 0.32 | 0.18 | 0.46 |  | 0.23 | 0.1 | 0.37 |
| H’ | Exotic species |  | 0.54 | 0.4 | 0.68 |  | 0.5 | 0.37 | 0.63 |
| FDis | All species |  | 0.26 | 0.23 | 0.3 |  | 0.24 | 0.21 | 0.27 |
| FDis | Native species |  | 0.07 | 0.04 | 0.11 |  | 0.05 | 0.02 | 0.08 |
| FDis | Exotic species |  | 0.17 | 0.13 | 0.21 |  | 0.15 | 0.11 | 0.18 |
